# Complete penile disassembly for isolated penopubic epispadias repair: The "Belgrade approach"

**DOI:** 10.1590/S1677-5538.IBJU.2025.0171

**Published:** 2025-05-05

**Authors:** Bruno Bucca, Borko Stojanovic, Marta Bizic, Marko Bencic, Slavica Pušica, Miroslav Djordjevic

**Affiliations:** 1 Sapienza Rome University Department of Maternal Childhood and Urological Sciences Rome Italy Department of Maternal Childhood and Urological Sciences, Sapienza Rome University, 00185 Rome, Italy; 2 Belgrade Center for Genitourinary Reconstructive Surgery Belgrade Serbia Belgrade Center for Genitourinary Reconstructive Surgery, 11000 Belgrade, Serbia; 3 University of Belgrade Faculty of Medicine Belgrade Serbia Faculty of Medicine, University of Belgrade, 11000 Belgrade, Serbia

## Abstract

**Introduction:**

Surgical treatment of epispadias has evolved significantly, from early tubularization techniques to modern penile disassembly approaches (1–3). Despite advancements, achieving urinary continence remains challenging and typically requiring multiple interventions (4).

**Purpose:**

The objective is to present complete penile disassembly (Belgrade) technique for primary epispadias repair.

Patient and Method: We present a case of isolated penopubic epispadias and severe dorsal curvature in 18-month-old boy. Meticulous dissection is conducted ventrally and dorsally to isolate the urethral plate and spongiosal tissues. The Buck's fascia is incised ventrally to isolate the neurovascular bundles, followed by complete separation of the corpora cavernosa from each other and from the glans. The urethral plate is dissected free, transposed ventrally, and tubularized over a catheter. Penile straightening and lengthening are achieved through internal rotation of the corpora cavernosa and dorsal corporotomy with skin grafting. Glans reconstruction is done. Reassemble of all entities is performed, followed by penile skin reconstruction.

**Results:**

At the three-month follow-up, the patient demonstrated satisfactory voiding with a good urinary stream, without evidence of urethral fistula or stricture. The cosmetic outcome was favorable, with no signs of recurrent curvature. The patient remains under vacuum device therapy, till 12 month after sugery.

**Conclusion:**

The Belgrade approach for isolated penopubic epispadias includes radical approach with complete disassembly. This one-stage repair enables correction of all deformities – penile lengthening and strengthening, urethroplasty, glansplasty and penile skin reconstruction, with good outcomes.

Available at: http://www.intbrazjurol.com.br/video-section/20250171_Bucca_et_al
